# Resting Network Plasticity Following Brain Injury

**DOI:** 10.1371/journal.pone.0008220

**Published:** 2009-12-14

**Authors:** Toru Nakamura, Frank G. Hillary, Bharat B. Biswal

**Affiliations:** 1 Department of Radiology, University of Medicine and Dentistry of New Jersey – New Jersey Medical School, Newark, New Jersey, United States of America; 2 Department of Psychology, Penn State University, University Park, Pennsylvania, United States of America; Indiana University, United States of America

## Abstract

The purpose of this study was to examine neural network properties at separate time-points during recovery from traumatic brain injury (TBI) using graph theory. Whole-brain analyses of the topological properties of the fMRI signal were conducted in 6 participants at 3 months and 6 months following severe TBI. Results revealed alterations of network properties including a change in the degree distribution, reduced overall strength in connectivity, and increased “small-worldness” from 3 months to 6 months post injury. The findings here indicate that, during recovery from injury, the strength but not the number of network connections diminishes, so that over the course of recovery, the network begins to approximate what is observed in healthy adults. These are the first data examining functional connectivity in a disrupted neural system during recovery.

## Introduction

### Advancing the understanding of traumatic brain injury via functional imaging

Traumatic brain injury (TBI) is a debilitating neurological disorder defined as an injury from an external source resulting in a period of altered consciousness and deficits in physical, cognitive, and/or psychosocial functioning. While examination of behavioral deficits associated with head trauma has a very long history, spanning over 60 years, only recently have the consequences of TBI received attention via functional imaging methods.

“Activation” studies using functional MRI and positron emission tomography have been used to examine TBI-related deficits in episodic memory [1], [2], working memory [3]–[7], and executive control [8]. While the results of these studies have generated working hypotheses regarding how plasticity is expressed in disrupted neural systems, discrete regions of interest and localized “activation” results have not been interpreted in the context of an integrated neural network.

There has been recent emphasis in studies using BOLD fMRI to approximate brain activity, to incorporate baseline or “resting” measurements of the BOLD signal. Systematic examination of baseline BOLD signal was first introduced by examining motor cortex [9] and has recently received significant attention resulting in demonstration of a discrete system of networks that are non-task or “default mode” [10], [11]. Emanating from these early findings has been a wellspring of studies examining resting brain states in the context of cognitive, sensory, and motor functioning. Most recently, these methods have been applied in cross-sectional work examining resting BOLD states in the clinical neurosciences.

Resting state fMRI has thus provided unique information about the behavior of voxels (or “networks”) in the absence of direct stimulation. What has not been examined in this relatively new literature is if resting states are plastic, and, in particular, if they are changing after neural disruption. The current study aims to document change in resting neural networks during recovery from brain injury by examining macro-level functional connectivity in the BOLD fMRI signal via *graph theory* (described below). To date, there has been no work using serial MRI to examine changes in neural connectivity during recovery from neurological insult and such methods may provide additional insight into how neural plasticity is expressed in the injured brain. Such analyses may offer insight into how networks adapt to neurological disruption. For example, it remains unclear if the neural recruitment observed almost universally in cross-sectional “activation” studies of working memory deficit is due to formal brain reorganization or is indicative of neural inefficiency during periods of cognitive challenge [12], [13]. What appears to be a critical element in making this determination is the nature of this neural recruitment over time and if the number of neural connections is altered during recovery. Activation studies in clinical samples can be methodologically challenging [14]–[17] and one potential method for examining how plasticity is expressed in the injured brain during recovery is to first document how resting networks are altered. Thus, it is an important aim to determine if, during recovery, networks become more elaborate, including the creation of additional connections, or if early recruitment of resources reflects use of available auxiliary networks, that later give way to diminished connectivity and greater neural efficiency. The whole-brain network analyses conducted here afford this opportunity.

### Graph theory and Neural networks

*Graph* theory provides a powerful framework for the mathematical treatment of complex systems such as neural networks in the brain [18]–[22]. The use of *graph* theory to date has led to important developments in how neural systems are understood, including small-world networks [18], [19], [22], scale-free network properties [23], “robustness for malfunction” [19], [24], and critical state in healthy subjects [25]. Furthermore, early application of this type of network analysis to the study of neurological disorders, such as Alzheimer's disease [26], [27] and schizophrenia [28], [29], has helped to characterize network abnormalities such as “neural disconnection”.

In the current study, *graph* theory is used to examine changes in whole brain resting connectivity during recovery from moderate and severe TBI. To do so, we analyze “ resting” data in order to characterize changing network properties during a time period now well established as a critical window for recovery of cognitive functioning in TBI [30], [31].

There are two over-arching goals in the current study. First, we aim to examine potential changes in resting connectivity during a period when behavioral recovery is known to occur after severe traumatic brain injury. To do so, resting data derived from working memory task data are analyzed at two time points early after injury. This approach affords the opportunity to observe the expression of neural plasticity during this critical window of recovery. Second, we use graph theory to examine changes in the resting BOLD response in order to examine whole-brain alterations in network connectivity over time. This approach represents a paradigm shift away from traditional cross-sectional work examining region-of-interest (ROI) changes associated with behavioral deficits. The inherent advantage to this approach is that whole-brain data analysis of “resting state data” effectively circumvents many of the methodological pitfalls associated with examining “activation” changes in clinical samples (e.g., task difficulty, performance inequalities) (see [12], [15]). Thus, the current study examines network change after injury via graph theory analyses in order to identify the how plasticity is expressed during recovery. Ultimately, these data may offer additional context for understanding the findings in the current cross-sectional literature using fMRI to examine deficits in working memory and rapid decision making.

## Results

### Strength of Functional Connectivity

**Figure 1:**
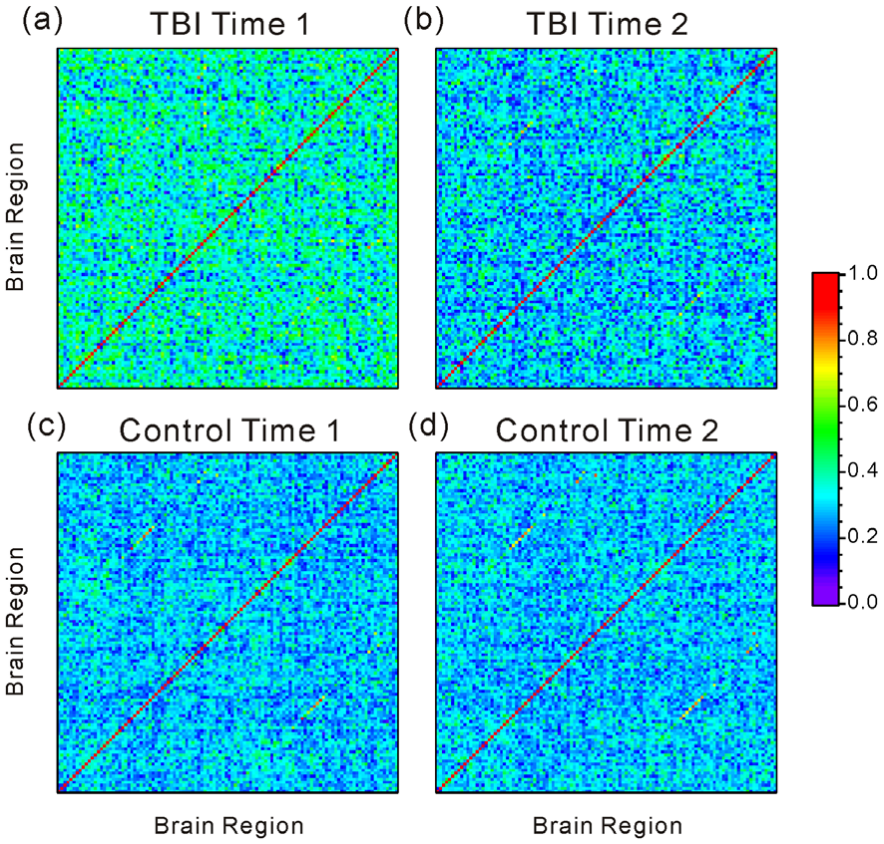
Mean absolute partial correlation matrix of TBI. The averaged absolute partial correlation matrix of TBI at (a) Time 1 and (b) Time 2, and Healthy subjects at (c) Time 1 and (d) Time 2. Both matrices consist of correlation coefficients of all pairs of 112 regions. The list of brain regions we used in this analysis are shown in Table S1. Each component in the matrix indicates the mean absolute strength of the functional connectivity between a pair of brain regions. The correlation coefficients are color coded.

The averaged functional connectivity matrices for Time 1 and Time 2 were calculated by averaging absolute correlation matrices in the TBI sample (Fig. 1). The strength of functional connectivity diminished from Time 1 to Time 2, more closely approximating the results observed in the HC sample.

**Figure 2:**
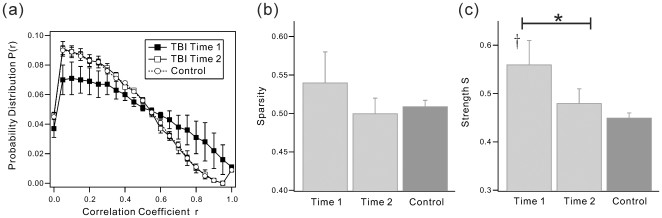
Properties of functional correlation matrix. (a) The group averaged probability distribution functions of absolute correlation coefficients with bin-width 0.05. (b) Sparsity and (c) Strength ***S*** of the networks from TBI Time 1, Time 2, and Control group. The value of group averaged mean correlation coefficient 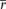 was 0.40±0.05 for TBI Time 1, 0.31±0.01 for TBI Time 2, and 0.30±0.03 for Control, respectively. * indicates significant difference between Time 1 and Time 2. † indicates significant change from control group.

Figure 2(a) shows group averaged probability distributions 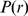 of absolute partial correlation coefficients for both groups. When comparing Time 1 and Time 2, lower correlation values ranging from *r* = 0.05 to 0.5 increased over time, but correlation values ranging from *r* = 0.6 to 0.95 decreased during recovery. The mean correlation coefficients were significantly different between time-points (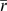 = 0.40±0.05 for Time 1 and 0.31±0.01 for Time 2), and values for Time 2 approximated those observed in the HC sample (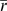 = 0.30±0.03).

Though the sparsity of the network showed a non-significant difference between time points (Fig. 2(b)), the strength ***S*** significantly decreased at Time 2 (Fig. 2(c)), closely approximating the results observed in healthy adults. These results indicate that the frequency of strong functional connectivity decreased during recovery, but the overall number of connections in the network remained relatively stable from Time 1 to Time 2. The specific values for all indices are summarized in Table 1.

**Table 1:** Functional Brain Network properties of “Un-weighted” network with threshold value *p*<0.05.

	*Sparsity*	*S*	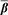	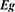	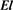	*L*	*C*	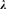	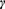	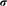
**TBI (Time 1)**	0.54±0.04	**0.56±0.05** * †	**3.65±0.41** * †	0.77±0.02	**0.83±0.02** †	1.47±0.04	**0.66±0.04** †	1.00±0.00	1.08±0.05	1.08±0.05
**TBI (Time 2)**	0.50±0.02	**0.48±0.03**	**4.58±0.18**	0.75±0.01	0.79±0.00	1.50±0.02	0.58±0.01	1.00±0.00	1.09±0.02	1.09±0.02
**Healthy**	0.51±0.01	**0.45±0.00**	**4.78±0.16**	0.75±0.00	**0.79±0.00**	1.49±0.01	**0.58±0.01**	1.00±0.00	1.07±0.00	1.07±0.00

* indicates significant difference between Time 1 and Time 2.

† indicates significant change from control group.

### Network Properties of Un-weighted Functional Connectivity Network

Table 1 summarizes network properties of un-weighted functional brain networks. All indices characterizing network statistical properties did not show any significant differences between time points. Local efficiency 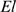 and clustering coefficient ***C*** were higher at Time 1 relative to HC.

### Network Properties of Weighted Functional Connectivity Network

**Figure 3:**
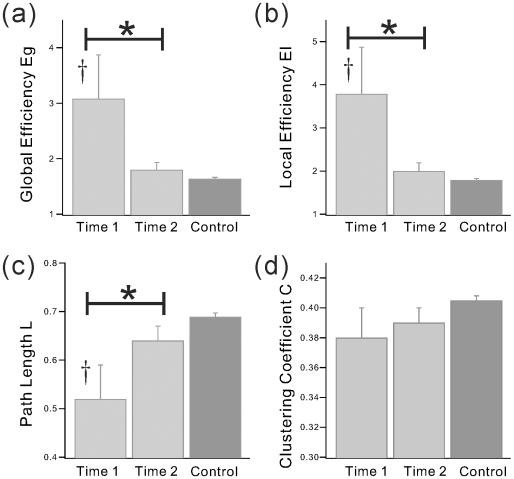
Functional Network properties of weighted networks (1). The weighted network properties; (a) Global efficiency 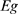, (b) Local efficiency 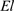, (c) Characteristic path length ***L***, and (d) Clustering coefficient ***C***, from TBI and Control groups. Here, the value of weight 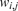 for each edge 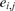 in the network was defined as 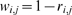, where 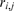 is the absolute partial correlation coefficient between region *i* and *j*. * indicates significant difference between Time 1 and Time 2. † indicates significant change from control group.

**Figure 4:**
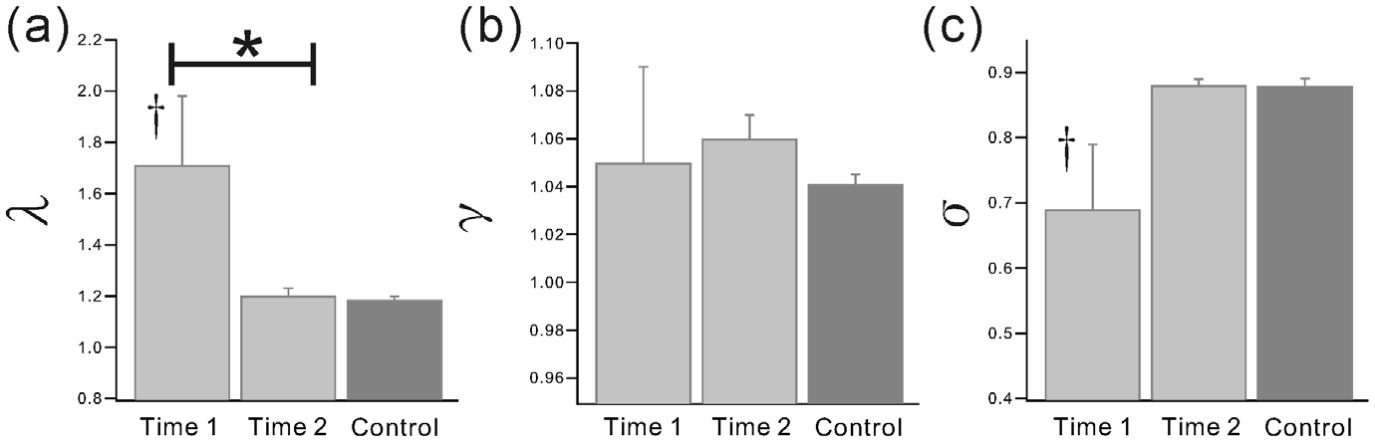
Functional Network properties of weighted networks (2). The weighted network properties; (a) 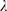, (b) 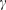, and (c) 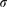. *indicates significant difference between Time 1 and Time 2. † indicates significant change from control group.

The correlation matrix revealed varying strengths in functional connectivity dependent upon the brain region pairings (see correlation matrices, Fig. 1) and the distributions of correlation coefficients was influenced by time point of measurement (see Fig 2(a)). These findings suggest that the strength of functional connectivity may be altered during recovery from TBI. Because un-weighted network analysis does not consider region-specific connectivity strengths and relies solely upon a binary description of connectivity (e.g., 1 or 0), emphasis in the current study was placed upon results from weighted networks. Figure 3 and Figure 4 show network indices of weighted functional connectivity network. The global efficiency 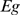 (Fig. 3(a)) and local efficiency 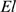 (Fig. 3(b)) showed significantly higher values at Time 1 compared with Time 2 and Control. These findings are consistent with an overall reduction in “strength” in the connected network. Because of its inverse relationship to 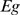, path length ***L*** (Fig. 3(c)) significantly increased during recovery. Thus, as network strength (and reciprocally global and local efficiency) decreased during recovery and these index changes from Time 1 to Time 2 became more consistent with healthy adults.

The results of indices which were normalized by producing randomized networks are shown in Fig 4. The components making up “small worldness”, 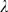 changed from Time 1 to Time 2, approximating what was observed in healthy adults over time (see Fig. 4(a)). The small-worldness index 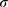 was significantly lower at Time 1 compare with Control, indicating the loss of small-worldness following injury. Of note, the two estimates of “length” in connectivity, path length ***L*** and 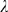, were significantly different in opposite directions. Because 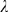 is a measure of path length standardized via the randomized networks, further discussion focuses on this finding. The specific values of all indices for weighted network analysis are summarized in Table 2.

**Table 2:** Functional Brain Network properties of “Weighted” network with threshold value *p*<0.05.

	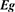	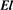	*L*	*C*	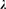	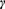	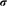
**TBI (Time 1)**	**3.08±0.80** * †	**3.79±1.08** * †	**0.52±0.07** * †	0.38±0.02	**1.71±0.27** * †	1.05±0.04	**0.69±0.10** †
**TBI (Time 2)**	**1.80±0.13**	**2.00±0.19**	**0.64±0.03**	0.39±0.01	**1.20±0.03**	1.06±0.01	0.88±0.01
**Healthy**	**1.64±0.03**	**1.79±0.03**	**0.69±0.01**	0.41±0.00	**1.19±0.01**	1.04±0.00	**0.88±0.01**

* indicates significant difference between Time 1 and Time 2.

† indicates significant change from control group.

One concern regarding network “weighting” is that findings may be at least partially attributable to statistical thresholding effects. When considering alternative statistical thresholds, the current results were observed to be quite robust; the results are not altered when comparing significant levels of *p*<0.05 and *p*<0.01 (see Tables S2 and S3).

### Degree Distribution in Functional Connectivity Network

**Figure 5:**
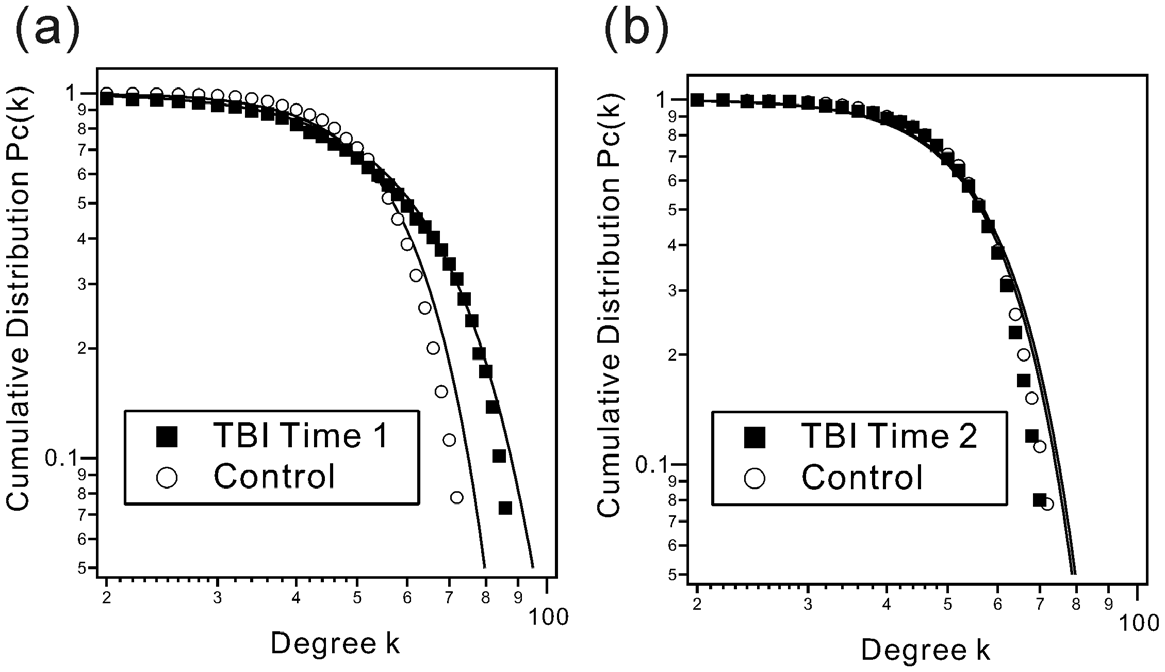
Cumulative degree distributions. The group averaged cumulative degree distributions 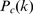 from (a) TBI at Time 1 and (b) Time 2 on double logarithmic scale. The solid curves are the best fitting stretched exponential function 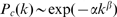 to the averaged cumulative degree distributions ranging from degree *k* = 30 to 80. The value of fitting parameter 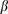 was 3.65±0.41 for TBI at Time 1 and 4.58±0.18 for Time 2, respectively. For Control group, the value of 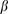 was 4.78±0.16.

For both groups the cumulative degree distribution 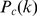 followed a non-Gaussian distribution with stretch-exponential-like heavy tails (Fig. 5). The distribution of Time 2 showed the similar decay as that of the HC group (Fig. 5(b)). By contrast, the distribution of Time 1 showed slower decay than that of Time 2 and the HC group, indicating an increase in the number of regions highly interconnected regions at Time 1 (Fig. 5(a)). Furthermore, the distribution of Time 1 was similar to the form of power-law with exponential decay 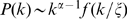, compared with Time 2 and the data derived from HCs.

In order to quantify change in the distributions from Time 1 to Time 2, the stretched exponential function 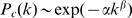 was fitted to each, using a range from degree *k* = 30 to 80. The resultant averaged fitting value of 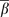 was 3.65±0.41 for Time 1 and 4.58±0.18 for Time 2 (Table 1). The value of 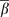 was 4.78±0.16 for Control group. The change in these distributions was significant between time points and Time 2 more closely approximating the shape of that observed in healthy adults.

## Discussion

We examined changes in functional connectivity at separate time points during a known period of recovery after TBI [30], [31]. To do so, we employed *graph* theory analyses to document changes in resting BOLD signal between time points. To the best of our knowledge, this is the first report examining functional connectivity during recovery from neurological insult using graph theory.

Un-weighted networks here revealed no significant changes between time points in neural networks during recovery. There is compelling evidence, however, that networks “weighted” according to a partial correlation matrix maintain important advantages over “un-weighted” data for understanding network characteristics [32]. That is, weighted networks maintain information not only about suprathreshold connections, but the magnitude of those connections which potentially offers greater sensitivity to network change during a period of functional recovery. For these reasons, the following discussion focuses on the results of weighted network analysis.

The current study analyzed “resting” data (as opposed to BOLD data associated with a known task) that are the result of a WM task. To provide context for understanding the resting connectivity data analyzed here, the “activation” findings achieved using a block design task revealed decreased involvement of relevant neural substrates (in particular prefrontal cortex) from Time 1 to time 2 [33], [34]. The findings here are consistent with what we have also observed during tasks of rapid decision making in TBI where task acclimation and faster reaction times result in diminished task-induced activation [34].

In a growing functional imaging literature examining deficits in clinical samples, there remains little work examining how neurological insult influences connectivity and information transfer between nodes. That is, important questions remain regarding how neural networks respond to disruption and how plasticity is expressed in these networks during recovery. Overall, the results here indicate that recovery from TBI is associated with three important changes in resting networks: 1) a reduction in the number of highly significant connections in the overall network (strength **S**), or reduced “cost”, but little to no change in the number of connections, or sparsity; 2) a transition of the degree distribution from a power law with exponential decay at three months post injury to a stretched exponential decay at six months post injury; 3) increasing “small-worldness” during recovery, but residually diminished small-worldness compared to the HC sample. These findings are highlighted below.

The degree distribution is a fundamental component of the indices that characterize a small-world network [20]. Again a “degree” is the connectedness of any node to the network and network distributions take any of several forms including Gaussian, which is commonly observed in random networks or non-Gaussian forms including distributions with a fat tail (e.g., scale free networks). In the current study, the distribution more closely approximated power-law with exponential decay at Time 1 in the TBI sample, indicative of greater flexibility and reduced restriction on network change [35]. This distribution shifted to becoming a stretched exponential decay distribution by Time 2 which more closely approximated what was observed in HCs (see Fig. 5).

Thus, changes from Time 1 to Time 2 reflect a change from power-law with exponential decay to a distribution indicative of a more restrictive network, such as stretched exponential decay, which maintains greater constraint on overall network plasticity [35]. From the viewpoint of statistical physics, such a transition in the tail of the degree distribution during recovery may be related to critical phenomena [35], [36] and, indeed, recent work has reported observing characteristics consistent with criticality in the brain [37], [38], [23]. Thus, this change in the degree distribution during recovery from neurological insult may operate to: 1) reduce “cost” in the network, 2) formalize the network, reducing its malleability and maximizing its efficiency. Earlier after injury, a distribution that more closely approximating power law might work to permit greater plasticity in order to accommodate challenge/disruption in the network. This early brain state may be neurally “expensive”, however, later giving way to a reduction in the number of highly significant connections and the overall strength of the network and increased small-worldness after recovery (i.e., Time 2). Overall, the current findings demonstrate that the degree distribution may be informative regarding the changing flexibility in global brain states.

In “activation” studies in the clinical working memory literature a nearly universal finding is that neurological insult results in recruitment of additional neural resources during periods of cognitive challenge (see [33]). In the current resting data, the change occurs in the context of consistent “sparsity”, indicating that the magnitude of the relationships between nodes is reduced but the number of connections remains stable. If it can be assumed that not only the number but also the nature of the connections is essentially maintained from Time 1 to Time 2, it appears that existing support networks are recruited to tolerate network disruption and that the demand for these resources diminishes after recovery. These findings complement what is observed in cross-sectional studies of TBI [3], [34], [5] and in healthy adults during periods of high task load [39]–[41] whereby increased involvement of neural resources may be highly dependent upon performance and does not represent fundamental changes in the network. That is, the alterations in neural connections observed during recovery may not signify formal brain reorganization (e.g., creation of novel connections). Instead these changes represent utilization of existing support resources early after neural disruption and this demand on auxiliary resources later remits resulting in a less costly network with greater neural efficiency.

The current data demonstrate that early after injury, resting networks may be composed of more highly significant connections, creating a network higher in local clusters with shorter path length and a degree distribution that approximates the power law with cut-off. One characteristic of this degree distribution is that it permits greater neural dynamics and flexibility for adjusting the strength of connections within the network. One interpretation is that, over time, recovery results in resting connectivity that is diminished in overall strength and increased small-worldness approximating what is observed in healthy adults. Thus, recovery following TBI does not appear to result in elaboration and permanent inclusion of additional connections to the network, which may have important implications for understanding the neural recruitment observed in activation studies to date.

While the current findings are the first to use graph theory and weighted network analysis to examine neural recovery in humans, this report is not without limitations. The most important shortcoming to the current study is the limited sample size, which does influence the generalizeability of the findings. Future work should endeavor to increase the sample size so that sample subtypes may be examined (e.g., injury severity, magnitude of behavioral improvement). Even so, there are two reasons why we believe the sample size here can support the conclusions made in this study. First, the effect of time on network analysis was large and, therefore observable (and statistically significant) within this small sample size. Second, for all indices, meaningful change from Time 1 to Time 2 resulted in networks more closely approximating those observed in the healthy adult sample. In sum, the current findings represent an important first look at how networks adapt to disruption during a critical window of recovery.

## Materials and Methods

### Participants

Participants recruited for the study included 8 healthy adult control participants (HCs) (3 females, ages 19–51) and 8 individuals (3 females) sustaining severe TBI between the ages of 19 and 55 underwent MRI scanning at separate time-points. Two subjects with TBI were later excluded following data collection due to significant head movement (n = 1) and poor compliance with task demands (n = 1). Clinical descriptive information regarding patient injury is available in Table 3. The severity of TBI was defined using the Glasgow Coma Scale (GCS) in the first 24 hours after injury [42] and GCS scores from 3–8 were considered “severe”. The BOLD fMRI data were collected at approximately 3 months and 6 months after resolution of posttraumatic amnesia; these 3-month and 6-month follow-up time-points are henceforth referred to as Time 1 and Time 2. Of note, subjects were selected based upon observable injury to prefrontal cortex which is known to subserve WM dysfunction, (see Fig. S1), thus providing the opportunity to directly examine the influence of disruption in neural networks related to the task. Prior to official enrollment in the study, all subjects signed IRB-approved informed consent forms approved by the Human Subjects Projection Office (HSPO) at Hershey Medical Center (HMC) in Hershey, PA. All study procedures complied with the HSPO office at HMC and with The Health Insurance Portability and Accountability Act (HIPAA) standards.

**Table 3:** Demographic, clinical and behavioral data for the subjects with TBI.

S#	G	Age	GCS	CT	Complications	Acute stay (days)
1	f	25	4	Bilateral SDH	None	16
2	m	39	6	L Frontal SDH, L IVH, DAI	None	21
3	m	21	3	Bi frontal contusion	Multiple facial fractures	32
4	f	56	14	L frontal contusion	None	5
5	f	19	11	L Temporal contusion	None	4
6	m	28	3	Bifrontal hematoma, SAH, left temporal SAH	None	12

L = left, SDH = subdural hematoma, IVH = intraventricular hemorrhage, DAI = diffuse axonal injury, SAH = subarachnoid hemorrhage, Acc = task accuracy during WM paradigm, RT = reaction time during fMRI WM task.

### Experimental Design

This study employed a simple non-verbal working memory task requiring delayed-response scenarios. This task primarily requires sustained visuo-spatial attention, working memory rehearsal, and speeded processing. The detail information regarding the cognitive task and data analysis methods are identical to those presented previously (see [33]). In brief, the task requires memory for included exposure to one, two, or four black and white images of male and female faces. This paradigm required the subject to examine a target slide for 3000 msecs. After a delay of 3000 msecs requiring focus on a fixation point, the subject is provided a final target stimulus, or a single face presented in one of four quadrants. At the time of presentation of the Target, the subject is required to make a yes/no decision about the identity (match/no match) and the location of a single face presented in one of the four quadrants. In this delayed response task, the subject is required to examine a Stimulus Slide for 3000 msecs and after a delay of 3000 msecs (fixation point), the subject made a determination regarding a final target stimulus (3000 msecs).

### Data Acquisition

For all scans, data were acquired using a Philips 3T system (Philips Medical Systems, Best, the Netherlands) in the Department of Radiology, Hershey Medical Center, Hershey, PA. High resolution anatomical images (MPRAGE) with isotropic spatial resolution of 1.2 mm×1.2 mm×1.2 mm were acquired. Other imaging parameters of the MPRAGE sequence consisted of: 468.45 ms/16.1 ms/18, TR/TE/flip angle, 250×200 mm^2^ FOV, and a 256×180 acquisition matrix. Imaging parameters for echo planar imaging (EPI) consisted of: 2000 ms/30 ms/89, TR/TE/flip angle, mm^2^ FOV, 256×180 acquisition matrix. In this block design paradigm, 166 images were obtained for each run for a total scanning time of 6 minutes and 32 seconds.

### Anatomical Parcellation and Pre-Processing

AFNI [43] was used to perform the following preprocessing steps, including motion correction, spatial smoothing, and mean-based intensity normalization. As a final preprocessing step, each individual's time series was spatially normalized by registration to the MNI152 template (Montreal Neurological Institute), with 2 mm^3^ resolution, using a 12 degrees-of-freedom affine transformation. Parcellation divides each cerebral hemisphere into 56 anatomical regions of interest (ROI). Regional mean time series were estimated for each individual by averaging the fMRI time series over all voxels in each of 112 regions. The resultant signals ware filtered with a bandpass filter ([0.01, 0.1] Hz).

Recently, Fox et. al reported that task-evoked activity are linearly superimposed on underling spontaneous activity (resting data) [44], [45]. This funding suggests that if the effect of task-induced activity is adequately removed from task-related design data, the remaining residual component should represent resting state data. The deterministic task-related brain activity can be properly modeled using a linear model, and can be removed by regression approaches. Here, we employed an orthogonalization method to remove task-related effects from intrinsic BOLD fluctuations [46].

### Estimation of Strength of Functional Connectivity

Functional connectivity is defined as the temporal correlations between spatially remote neurophysiological events [9], [47]–[49]. The correlation between a given pair of regions is typically used to index functional connectivity [19], [23], [28], [22] and the resultant pair-wise correlation matrices ***R*** are then thresholded to generate a statistically significant functional connectivity network.

In this study, the partial correlation coefficients 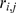 were used as a measure of functional connectivity between given pairs of BOLD signal from regions *i* and *j* (*i*, *j* = 1,2,…,*M*; here *M* is a number of ROIs). The partial correlation matrix ***R*** (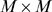) is a symmetric matrix in which each off-diagonal element is the correlation coefficient between a pair of regions after filtering out effects of all other brain regions. The procedure for obtaining partial correlation values here is consistent with other work [28], [22].

### Construction of Functional Connectivity Network

For the current data, construction of a functional connectivity network was achieved by testing the null hypothesis that the partial correlation coefficient 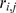 was significantly different from zero between any region pairs [50]. Two regions were considered *functionally connected* if their partial correlation coefficient was significant at *p*<0.05 level, resulting in an adjacency matrix *A* containing information about the connectivity structure of the network. From resultant matrix, the sparsity, which is the connection density in the network, and the strength ***S***, which is an average of all significant correlation coefficients and a measure of expensiveness in the network, were evaluated.

For the purposes of this study, both weighted and un-weighted networks were examined. In constructing un-weighted networks, each component of the adjacency matrix was labeled 1 if a corresponding 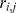 was significant, and 0 in other cases (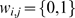). For weighted networks, a number of potential “weight” definitions can be used. Consistent with previous work using graph theory to examine neural systems [18], networks were defined with the definition 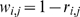 (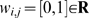) if the corresponding 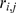 was significant and 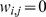 if it was non-significant. Such analysis results in adjacency matrices: *graph* 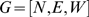 consisting of a set of nodes 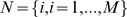 (brain regions), a set of edges 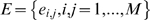 (significant connectivities), and a set of associating weights 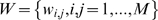 between node *i* and *j*. Of note, alternative weight definitions such as 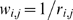 have been applied to examine other networks (e.g., world wide web) [51], [52]. This weight definition was used in the current data and the results were largely similar to those presented in this manuscript. We have chosen to focus here on the results using 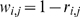 as a weight definition because of its prior use in neural systems.

### Network Properties: Path Length and Clustering Coefficient

In *Graph* theory, the clustering coefficient ***C***, and characteristic path length ***L*** of any *graph* ***G*** are key indices characterizing the statistical properties of a network [53]. For weighted networks the shortest path length 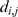 between two nodes *i* and *j* is the smallest sum of the weights for all the possible paths from node *i* to node *j*. The path length ***L*** of a *graph* ***G*** is the mean of the shortest path lengths over all possible pairs of nodes:
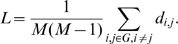

***L*** thus represents the extent and average connectivity or overall routing efficiency of a network.

The clustering coefficient ***C*** *i* of a node *i* with degree 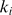 is calculated as the geometric average of subgraph weights [54]:
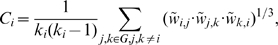
where 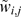 is the normalized weight by the largest value of the weight in the network 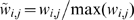. The clustering coefficient ***C*** of the network is an average of ***C*** *i* over all nodes. The clustering coefficient is an index of local structure, and has been interpreted as a measure of resistance to failure and the extent of the local density or cliquishness of the network.

In random networks, characteristic path length 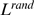 is typically short, and clustering coefficient 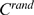 is typically small [55], [53]. In small-world networks, by definition, we expect the ratio 
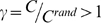
 and the ratio 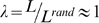 [53]. A scalar summary of small-worldness can be defined as the ratio 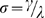, which is typically >1 for a small world network, or greater than random clustering and near random path length [19], [56]. In order to obtain random networks, we used a surrogate method where generated networks preserve the same number of nodes, edges, and degree (the degree of each node 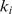 is the number of nodes directly connected to the node *i* distribution as the real network) [57]. For all data sets here, including each session per subject, 100 random networks were generated for each network and the averaged indices 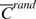 and 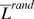 were calculated.

### Network Properties: Global and Local Efficiency

Application of *Graph theory* also provides the opportunity to examine network efficiency on global and local scales [18], [51], [52], [58]. The global efficiency 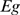 of a *graph* ***G*** is defined as;
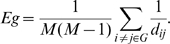
The global efficiency is an indicator of global efficiency of parallel information transfer in the network [18], [51], [52].

The local efficiency of each node 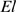 can be defined as follows:
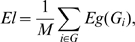
where ***G*** *i* is a set of nodes directly connecting to the *i*th node [18], [51], [52], [58]. The measure is a mean local efficiency of information transfer in the immediate neighborhood of each node [18]. Note that both 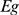 and 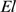 can be calculated for both un-weighted and weighted networks as well as path length and clustering coefficient.

### Network Properties: Degree Distribution

The degree distribution likely represents the most fundamental component of the various indices that characterize a graph [20]. The degree of each node 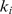 is defined as the number of nodes directly connected to the node *i* in the network, so the degree distribution 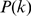 is the observed distribution of the number of direct connections of nodes in the network. Here we use a cumulative distribution 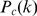 in the place of 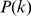. The degree distribution 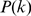 furthermore classifies small-world networks into different structural classes including: (a) scale-free networks, characterized by a degree distribution that decays as a power law, i.e 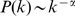, (b) broad-scale networks, characterized by a degree distribution that has a power law regime followed by a sharp cutoff 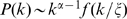, and (c) single-scale networks characterized by a degree distribution with a fast decaying tail [35].

### Statistics

Wilcoxon matched-pairs test was used to test significant differences between different time points. For Control group there were no significant differences in any network indices between point Time 1 and Time 2, thus we used the averaged values of both points for all indices to increase reliability in the observed networks. Dannet's test was used to determine the differences in network indices between the TBI sample and the Healthy Control (HC) sample. In the data presented here, error bars indicate the standard error of mean and an asterisk in the graphs indicates a significant difference between time points.

## Supporting Information

Figure S1 MR images of TBI. Axial MR images providing examples of the types of discrete frontal lesions occurring in this sample.
(1.09 MB TIF)

Table S1 The list of region name for Left/Right Hemisphere
(0.04 MB DOC)

Table S2 Functional Brain Network properties of un-weighted network with threshold value p<0.01
(0.03 MB DOC)

Table S3 Functional Brain Network properties of weighted network with weight definition w_ij_ = 1−r_ij_ and threshold value p<0.01
(0.04 MB DOC)
