# Supplementary figures and images for: Resting Network Plasticity Following Brain Injury
Source: PLoS One. 2009 Dec 14;4(12):e8220. doi: 10.1371/journal.pone.0008220 (PMC2788622; doi:10.1371/journal.pone.0008220)

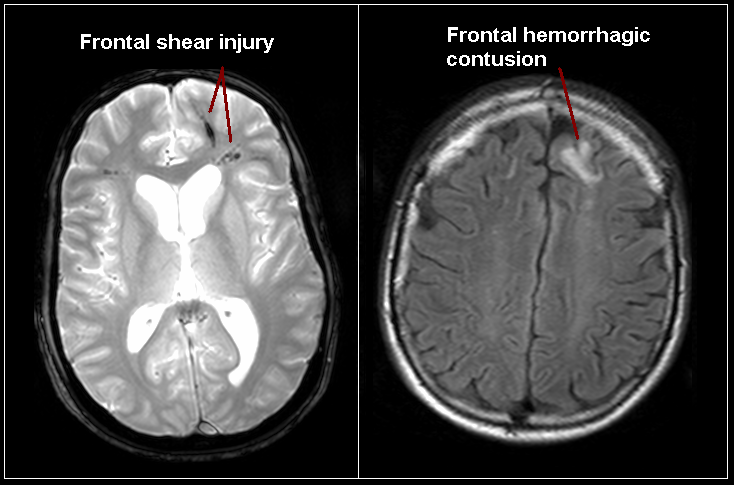

Supplement: Figure S1 — MR images of TBI. Axial MR images providing examples of the types of discrete frontal lesions occurring in this sample. (1.09 MB TIF) [file pone.0008220.s001.tif]
