# Supplementary material for: Resting Network Plasticity Following Brain Injury
Source: PLoS One. 2009 Dec 14;4(12):e8220. doi: 10.1371/journal.pone.0008220 (PMC2788622; doi:10.1371/journal.pone.0008220)
Supplement: Table S1 — The list of region name for Left/Right Hemisphere (0.04 MB DOC) [file pone.0008220.s002.doc]

**Table S1: The list of region name for Left/Right Hemisphere**

| **Region name** | |
| --- | --- |
| Frontal Pole | Cingulate Gyrus, Anterior |
| Insula | Cingulate Gyrus, Posterior |
| Superior Frontal Gyrus | Precuneous Cortex |
| Middle Frontal Gyrus | Cuneal Cortex |
| Inferior Frontal Gyrus, Pars Triangularis | Frontal Orbital Cortex |
| Inferior Frontal Gyrus, Pars Opercularis | Parahippocampal Gyrus, Anterior |
| Precentral Gyrus | Parahippocampal Gyrus, Posterior |
| Temporal Pole | Lingual Gyrus |
| Superior Temporal Gyrus, Anterior | Temporal Fusiform Cortex, Anterior |
| Superior Temporal Gyrus, Posterior | Temporal Fusiform Cortex, Posterior |
| Middle Temporal Gyrus, Anterior | Temporal Occiital Fusiform Cortex |
| Middle Temporal Gyrus, Posterior | Occiital Fusiform Gyrus |
| Middle Temporal Gyrus , temporooccipital part | Frontal Operculum Cortex |
| Inferior Temporal Gyrus, Anterior | Central Opercular Cortex |
| Inferior Temporal Gyrus, Posterior | Parietal Operculum Cortex |
| Inferior Temporal Gyrus, temporooccipital part | Planum Polare |
| Postcentral Gyrus | Heschl's Gyrus |
| Superior Parietal Lobule | Planum Temporale |
| Supramarginal Gyrus, Anterior | Supracalcarine Cortex |
| Supramarginal Gyrus, Posterior | Occipital Pole |
| Angular Gyrus | Thalamus |
| Laterial Occipital Cortex, Superior | Caudate |
| Lateral Occipital Cortex, Inferior | Putamen |
| Intracalcarine Cortex | Pallidum |
| Frontal Medial Cortex | Brainstem |
| Juxtapositional Lobule Cortex  (formerly Supplementary Motor Cortex) | Hippocampus |
| Subcallosal Cortex | Amygdala |
| Paracingulate Gyrus | Accumbens |

The ROIs listed above were extracted from each hemisphere
