# Supplementary material for: Resting Network Plasticity Following Brain Injury
Source: PLoS One. 2009 Dec 14;4(12):e8220. doi: 10.1371/journal.pone.0008220 (PMC2788622; doi:10.1371/journal.pone.0008220)
Supplement: Table S2 — Functional Brain Network properties of un-weighted network with threshold value p<0.01 (0.03 MB DOC) [file pone.0008220.s003.doc]

**Table S2: Functional Brain Network properties of un-weighted network with threshold value *p***<0.01.

|  | $Eg$ | $El$ | ***L*** | ***C*** | $\lambda$ | $\gamma$ | $\sigma$ |
| --- | --- | --- | --- | --- | --- | --- | --- |
| **TBI (Time 1)** | 0.70±0.02 | **0.81±0.02†** | 1.61±0.05 | **0.62±0.05†** | 1.01±0.01 | 1.20±0.11 | 1.18±0.10 |
| **TBI (Time 2)** | 0.69±0.01 | 0.76±0.00 | 1.63±0.02 | 0.53±0.01 | 1.00±0.00 | 1.26±0.05 | 1.26±0.05 |
| **Healthy** | 0.69±0.00 | **0.76±0.00** | 1.62±0.01 | **0.52±0.01** | 1.00±0.00 | 1.20±0.01 | 1.20±0.01 |

† indicates significant change from control group.
