# Supplementary material for: Resting Network Plasticity Following Brain Injury
Source: PLoS One. 2009 Dec 14;4(12):e8220. doi: 10.1371/journal.pone.0008220 (PMC2788622; doi:10.1371/journal.pone.0008220)
Supplement: Table S3 — Functional Brain Network properties of weighted network with weight definition wij = 1−rij and threshold value p<0.01 (0.04 MB DOC) [file pone.0008220.s004.doc]

**Table S3: Functional Brain Network properties of weighted network with weight definition$w_{ij}=1-r_{ij}$ and threshold value *p*<0.01.**

|  | $Eg$ | $El$ | ***L*** | ***C*** | $\lambda$ | $\gamma$ | $\sigma$ |
| --- | --- | --- | --- | --- | --- | --- | --- |
| **TBI (Time 1)** | **3.06±0.80＊†** | **4.11±1.18＊†** | **0.53±0.07＊†** | 0.36±0.01 | **1.69±0.25＊†** | 1.16±0.10 | 0.77±0.12 |
| **TBI (Time 2)** | **1.78±0.13** | **2.13± 0.23** | **0.65±0.03** | 0.36±0.01 | **1.21±0.03** | 1.21±0.05 | 1.00±0.02 |
| **Healthy** | **1.62±0.03** | **1.89±0.04** | **0.70±0.01** | 0.37±0.00 | **1.20±0.01** | 1.15±0.01 | 0.97±0.02 |

＊indicates significant difference between Time 1 and Time 2. † indicates significant change from control group.
